# Investigation of the Combined Impact of Zinc Oxide Nanoparticles and Bacterial Cellulose Nanocrystals on the Bond Strength to Dentin and Fracture Resistance of Resin‐Modified Glass Ionomer Cement in Primary Molars

**DOI:** 10.1002/cre2.70224

**Published:** 2025-09-19

**Authors:** Ali Nozari, Fatemeh Parvizi, Zahra Jowkar, Farnaz Haji Abbas Oghli, Hosein Askari, Seyed Ahmadreza Hamidi

**Affiliations:** ^1^ Department of Pediatric Dentistry, School of Dentistry Shiraz University of Medical Sciences Shiraz Iran; ^2^ Oral and Dental Disease Research Center, Department of Operative Dentistry, School of Dentistry Shiraz University of Medical Sciences Shiraz Iran; ^3^ Department of Oral and Maxillofacial Surgery, School of Dentistry Shiraz University of Medical Sciences Shiraz Iran

**Keywords:** bacterial cellulose nanocrystals, fracture resistance, mesoporous zinc oxide nanoparticles, microshear bond strength

## Abstract

**Objectives:**

This study aimed to evaluate the microshear bond strength (µSBS) of resin‐modified glass ionomer cement (RMGIC) to primary dentin and the fracture resistance of primary molars restored with RMGIC, with and without the incorporation of mesoporous zinc oxide nanoparticles (ZnO NPs) and bacterial cellulose nanocrystals (BCNCs).

**Materials and Methods:**

A total of 100 extracted primary mandibular second molars were divided into two tests: the µSBS test (40 teeth) and the fracture resistance test (60 teeth). The µSBS test included four groups: (1) RMGIC (control), (2) RMGIC + 5 wt.% mesoporous ZnO NPs, (3) RMGIC + 1 wt.% BCNCs, and (4) RMGIC + 5 wt.% mesoporous ZnO NPs and 1 wt.% BCNCs. The fracture resistance test included these groups along with an intact teeth group (positive control) and a prepared but unrestored teeth group (negative control). A universal testing machine was used for all mechanical tests.

**Results:**

The RMGIC + 1 wt.% BCNCs group exhibited the highest µSBS (6.35 ± 1.98 MPa), significantly surpassing the control and other experimental groups (*p* < 0.001). For fracture resistance, the negative control had the lowest value (422.70 ± 44.50 N, *p* < 0.05), while the positive control had the highest, significantly outperforming all groups except RMGIC + 1 wt.% BCNCs (*p* > 0.05). The RMGIC + 1 wt.% BCNCs group (1280.40 ± 340.87 N) demonstrated significantly greater fracture resistance than both RMGIC and RMGIC + 5 wt.% mesoporous ZnO (*p* < 0.05).

**Conclusions:**

Incorporating 1 wt.% BCNCs into RMGIC significantly enhanced both microshear bond strength and fracture resistance, leading to a higher proportion of restorable fractures. The positive correlation between bond strength and fracture resistance suggests that BCNCs‐modified RMGIC is a promising restorative material for improving durability in primary molars.

## 1 Introduction

Glass ionomer cements (GICs) are widely used restorative materials in pediatric dentistry. They offer several advantages, including fluoride release, biocompatibility, rapid cavity filling, and chemical adhesion to tooth structures (Pargaonkar et al. 2018). However, their clinical performance is often limited by weak mechanical properties, such as elastic deformation under masticatory forces, low fracture resistance, reduced flexural strength, and susceptibility to desiccation (Oznurhan and Ozturk 2020). To address these limitations, various strategies have focused on modifying GIC composition and incorporating reinforcing fillers (Menezes‐Silva et al. 2019; Jowkar et al. 2019; Sharafeddin et al. 2019).

Resin‐modified glass ionomer cements (RMGICs) improve upon conventional GICs by providing greater fracture toughness, hardness, flexural strength, wear resistance, and tensile strength (Rêgo et al. 2022; Malhotra et al. 2022). The added resin also shortens setting time, reduces moisture sensitivity, extends working time, enhances translucency, and improves esthetics (Rêgo et al. 2022; Malhotra et al. 2022).

In restorative dentistry, controlling bacterial colonization after caries removal is crucial, as bacteria can compromise restoration durability. Incorporating antibacterial agents helps prevent infiltration, microbial growth, and recurrent caries (Jowkar et al. 2019; Moradpoor et al. 2021). Moreover, evaluating the bond strength, marginal adaptation, and fracture resistance of different restorative materials to various dental substrates remains crucial for ensuring long‐term clinical success in restorative dentistry (Shafiei et al. 2017, 2014, 2020).

Zinc oxide (ZnO) is widely recognized for its antimicrobial properties, being cost‐effective, chemically stable, and safe (Moradpoor et al. 2021; Jowkar et al. 2020). ZnO disrupts bacterial membranes and enzyme activity by binding to bacterial cells (Moradpoor et al. 2021). ZnO nanoparticles (ZnO NPs) enhance these antibacterial effects, penetrate dentinal tubules more effectively, and inhibit biofilm formation by *S. mutans* and *Lactobacillus* when incorporated into dental materials (Moradpoor et al. 2021; Arun et al. 2020). Additionally, ZnO NPs can improve bond strength in enamel and dentin without compromising adhesion (Jowkar et al. 2018).

Mesoporous materials, with pore sizes of 2–50 nm, have gained attention in medicine and dentistry due to their tunable pores, high surface area, biocompatibility, and non‐toxicity (Yan et al. 2017). Their structure can be easily modified, and synthesis techniques allow optimization of composition, porosity, and crystallinity (Jowkar et al. 2025). Mesoporous ZnO NPs, with large surface area and antibacterial properties, show potential for diverse therapeutic applications (Yan et al. 2017; Jowkar et al. 2024).

A recent strategy to enhance RMGICs involves incorporating cellulose nanocrystals (CNCs) (Silva et al. 2013). Bacterial CNCs (BCNCs), produced by *Gluconacetobacter xylinum*, form a 3D network of microfibrils. Compared to plant cellulose, BCNCs have higher crystallinity, superior water retention, and greater mechanical strength in wet conditions (Gea et al. 2011; Gelin et al. 2007). They are biocompatible, lightweight, biodegradable, well‐dispersed, and form stable scaffolds (Cañas‐Gutiérrez et al. 2020).

Nano‐sized materials, such as mesoporous ZnO NPs, exhibit enhanced antibacterial properties compared to bulk forms due to their high surface area‐to‐volume ratio, making them promising nanofillers for RMGICs (Katoch et al. 2024). BCNCs, with high mechanical strength, can reinforce RMGICs. While adding nanofillers like BCNCs or mesoporous ZnO NPs can improve mechanical and antibacterial properties, maintaining bond strength and overall performance is essential. The combined effects of mesoporous ZnO NPs and BCNCs on bond strength and fracture resistance in primary molars restored with RMGIC remain unclear. Therefore, this study aimed to evaluate and compare the microshear bond strength (µSBS) of RMGIC to primary dentin and the fracture resistance of primary molars restored with RMGIC, with and without mesoporous ZnO NPs and BCNCs. The null hypothesis proposed no significant differences in bond strength or fracture resistance with or without these additives.

## 2 Materials and Methods

The study obtained 100 human primary mandibular second molars of similar size with intact facial and lingual surfaces, following approval from the Research and Ethics Committee of Shiraz University of Medical Sciences (Protocol #IR.SUMS.DENTAL. REC.1403.048). Moreover, all methods were carried out in accordance with the Declaration of Helsinki. These teeth were extracted for orthodontic reasons. Parents received detailed information about the study's purpose and the use of extracted teeth, providing written consent. The teeth were obtained from children between the ages of 4 and 6, with physiological root resorption limited to no more than one‐third of the root length. For consistency, teeth of similar size were selected by measuring their buccal and lingual crown dimensions (height and width) using a digital caliper with 0.01 mm accuracy (Pella Inc., Redding, CA, USA). The mesiodistal dimension averaged 10.45 mm, while the buccolingual dimension was approximately 9.33 mm. An experienced operator, blinded to the study conditions, performed all experimental procedures. Each tooth was inspected under a stereomicroscope (Carl Zeiss Inc., Oberkochen, Germany) at 20× magnification to ensure there were no cracks, fractures, wear, carious defects, or prior restorations. Soft tissue remnants were carefully removed, and the teeth were disinfected in a 0.1% chloramine T solution for 48 h. Following disinfection, they were stored in distilled water at 4°C for a maximum of 1 month before use.

**Figure 1:**
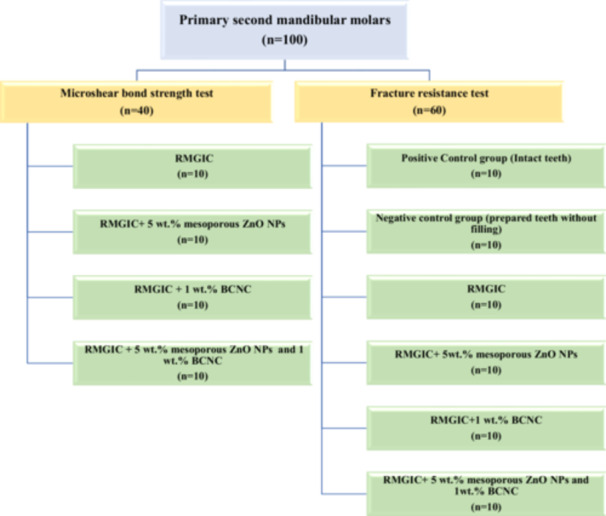
Schematic representation of the study design. (Abbreviations: BCNCs – bacterial cellulose nanocrystals; RMGIC – resin‐modified glass ionomer cement; ZnO NPs – zinc oxide nanoparticles.).

Figure 1 presents a graphical overview of the study groups and their associated protocols. This study incorporated two types of fillers into RMGIC. The first was mesoporous ZnO NPs, which were prepared and characterized according to a method described in a previous study (Jowkar et al. 2024). The other fillers were BCNCs (Nano Novin Polymer Co.; Gorgan, Golestan, Iran), derived from bacterial cellulose extracted from the *Gluconacetobacter* genus. Both mesoporous ZnO NPs and BCNCs were precisely measured to 0.001 g using a digital scale (GR‐3000, A & D CL Toshiba, Tokyo, Japan) and mixed with the pre‐weighed RMGIC powder (Fuji II, GC, Tokyo, Japan), which contained 95 wt.% fluoroaluminosilicate glass (amorphous) and 5 wt.% polyacrylic acid, in the specified concentrations for each group as detailed in the following sections.

### 2.1 Sample Size Calculation

In this study, four groups of samples were prepared for µSBS testing, while six additional groups were produced for fracture resistance evaluation. The sample size was calculated using G*Power 3.1 software (Heinrich Hein University, Dusseldorf, Germany) before the study began. The calculation was based on a comparison of two mean values (± standard deviations) from prior research, which were 2.09 ± 1.80 and 7.17 ± 2.14 (Moradian et al. 2021a). With a type I error (α) of 0.04 and a target power of 80%, the analysis recommended a minimum of four samples per subgroup. To account for potential dropouts and ensure adequate statistical power, the sample size was increased to 10 specimens per subgroup.

### 2.2 Sample Preparation for Microshear Bond Strength Test

Forty randomly selected primary second molar teeth with intact occlusal surfaces were prepared for the μSBS test. The occlusal enamel and upper dentin layers of the samples were carefully removed using a water‐cooled low‐speed cutting machine (Mecatome T201 A, Presi, Grenoble, France) to expose the dentin surface. To ensure uniformity, the prepared surfaces were polished for 20 s under running water using 600‐grit silicon carbide paper, creating a standardized smear layer. The specimens were then thoroughly rinsed and dried with an air‐water syringe. Next, the roots were sectioned 1 mm below the cementoenamel junction, and each tooth was mounted in acrylic resin (Acropars; Marlik Co., Tehran, Iran) with the dentin surface aligned parallel to the base of the mold for consistency in testing.

Forty well‐prepared specimens were divided into four groups, with each group consisting of 10 samples for μSBS testing as follows:

Group 1: RMGIC

Group 2: RMGIC + 5 wt.% mesoporous ZnO NPs

Group 3: RMGIC + 1 wt.% BCNCs

Group 4: RMGIC + 5 wt.% mesoporous ZnO NPs and 1 wt.% BCNCs

In group 1 (control group, *n* = 10), RMGIC restorations were prepared by mixing one scoop of control RMGIC powder (Fuji II LC Gold A2; GC, Tokyo, Japan) with two drops of liquid for 25 s, following the manufacturer's instructions to achieve a powder‐to‐liquid ratio of 3.2:1 by weight. For group 2 (RMGIC + 5 wt.% mesoporous ZnO NPs, *n* = 10), the composition consisted of 95 wt.% RMGIC and 5 wt.% ZnO NPs. In Group 3 (RMGIC + 1 wt.% BCNCs, *n* = 10), the mixture contained 99 wt.% RMGIC and 1 wt.% BCNCs. Group 4 (RMGIC + 5 wt.% mesoporous ZnO NPs and 1 wt.% BCNCs, *n* = 10) comprised 94 wt.% RMGIC, along with 5 wt.% mesoporous ZnO NPs and 1 wt.% BCNCs.

For groups 2, 3, and 4, the RMGIC powder was combined with mesoporous ZnO NPs and/or BCNCs and manually mixed to ensure uniform distribution. This mixture was then placed into amalgam capsules and blended for 20 s using an amalgamator (Ultramat 2, SDI Limited, Victoria, Australia). Finally, the blended powder was mixed with the RMGIC liquid (Fuji II, GC, Tokyo, Japan) to prepare the experimental materials for each group.

**Figure 2:**
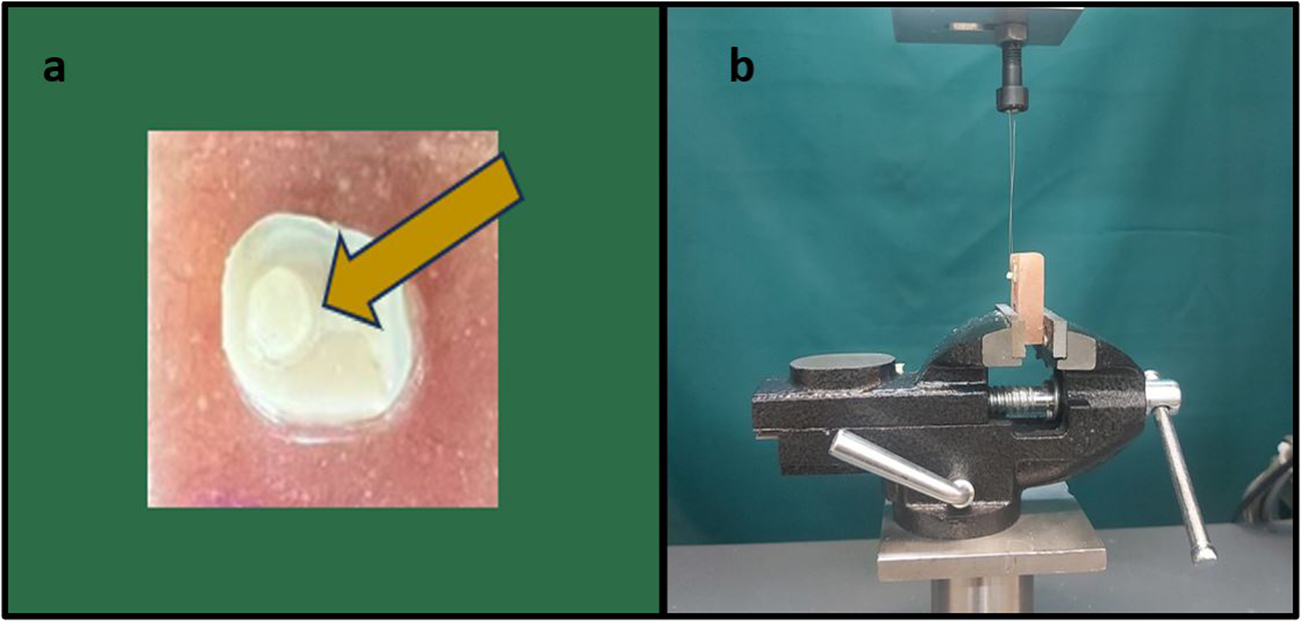
(a) A prepared specimen after bonding with resin‐modified glass ionomer cement (RMGIC), with arrows pointing to the RMGIC micro‐cylinder bonded to the dentin of the specimen. (b) Bonded specimen positioned under the universal testing machine for the microshear bond strength test, with a stainless‐steel ligature wire wrapped around the base of the RMGIC micro‐cylinder.

A hollow cylinder, approximately 0.5 mm in height and with an internal diameter of 0.7 mm, was cut from microbore Tygon tubing (R‐3603, Norton Performance Plastics Co, Cleveland, OH, USA) and positioned on the prepared dentin surface of each sample. The surface was outlined by adhesive tape with a punched hole at the center of the flattened dentin area of each sample. The hollow cylinder was then filled with RMGIC of each experimental group according to the manufacturer's instructions (Figure 2a). A mylar matrix strip was placed over the top surface of the mold filled with RMGIC from each experimental group. The top surface of each sample was then light‐cured for 40 s using an LED light‐curing unit (Blue Lex LD‐105; Monitex, Taipei, Taiwan) with a light intensity of 1500 mW/cm² and a wavelength range of 440–480 nm, following the manufacturer's instructions. The curing tip was kept 1 mm from the upper surface of the sample. Once the fabrication process was finished, the samples were stored in water at 37°C for 24 h to allow the material to set fully. Afterward, the Mylar strips and the Tygon tubings were carefully removed using a scalpel. A shear force was applied parallel to the bonded interface during the microshear bond strength test using a universal testing machine at a crosshead speed of 1 mm/min, with the force transmitted through a wire wrapped around the RMGIC until failure occurred (Figure 2b). The μSBS in Megapascals (MPa) for each sample was calculated by dividing the failure load by the bonded surface area.

Failure mode analysis:

Following the bond strength measurements, each fractured specimen was examined under a stereomicroscope (Carl Zeiss Inc., Oberkochen, Germany) at 40x magnification to assess the failure mode. The failure types were classified as:

A. Adhesive failure (at the interface between dentin and RMGIC)

B. Cohesive failure (within the RMGIC material or dentin)

C. Mixed failure (a combination of adhesive and cohesive failures) (El‐Deeb and Mobarak 2018).

### 2.3 Sample Preparation for Fracture Resistance Test

**Figure 3:**
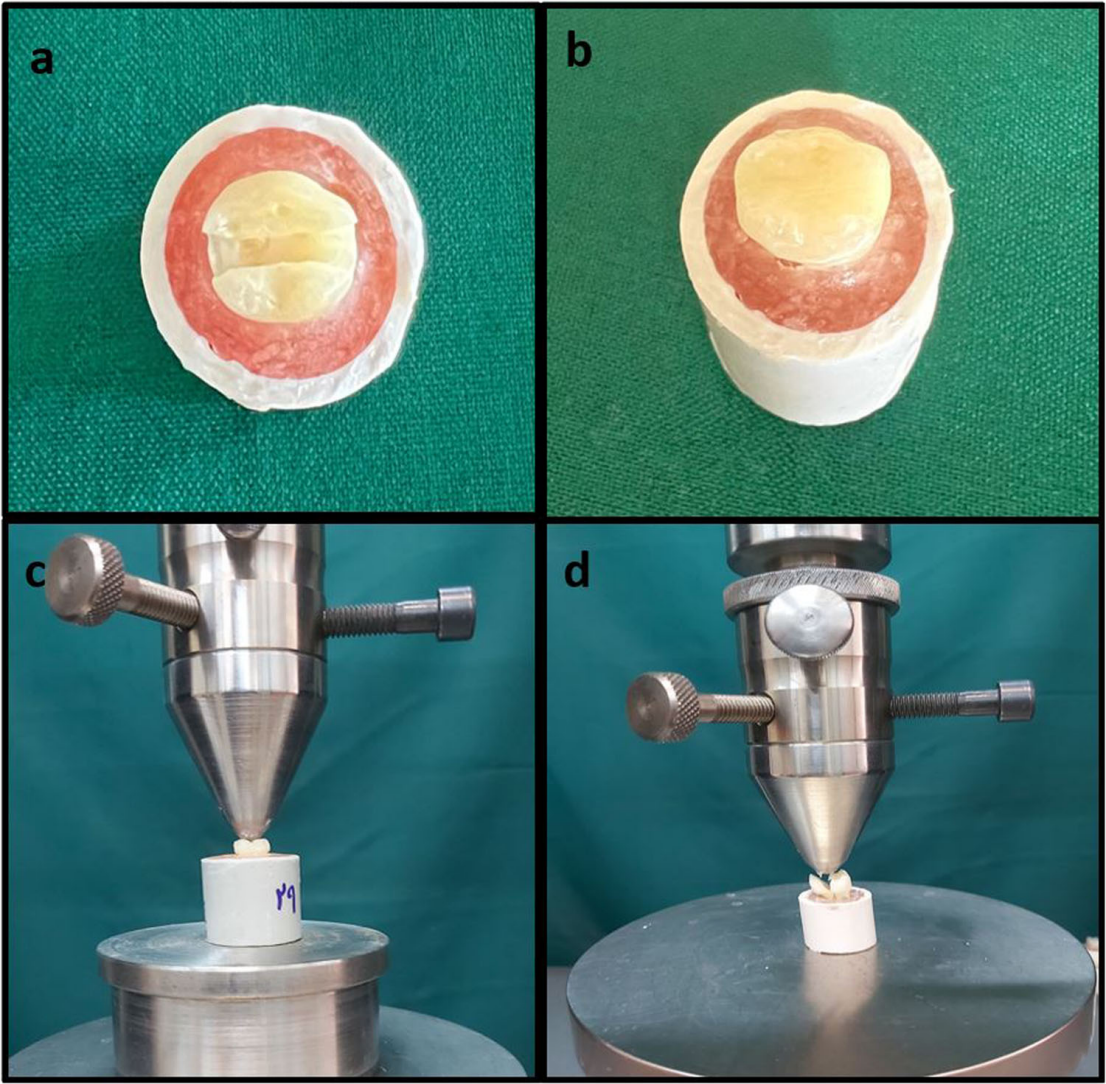
Step‐by‐step procedure for the fracture resistance test. (a) Each prepared molar was embedded in acrylic resin with its long axis positioned vertically to the horizontal plane. (b) The prepared cavity was restored using resin‐modified glass ionomer cement (RMGIC). (c) A steel tip (4 mm diameter) was mounted on the upper movable head of the testing device, applying a gradually increasing compressive force along the tooth's long axis. (d) The force was directed at the central fossa until fracture occurred.

Sixty teeth were randomly selected from the collected specimens for fracture resistance testing. The tooth preparation protocol for the fracture resistance test followed the methodology described in a prior study (Topçuoğlu and Topçuoğlu 2023). Each tooth was mounted in autopolymerizing acrylic resin (Acropars; Marlik Co., Tehran, Iran), positioning the resin 1 mm below the cemento‐enamel junction (CEJ). Each molar was positioned in acrylic resin with its long axis oriented vertically to the horizontal plane, ensuring that the buccal and lingual cusps were aligned on the same plane to achieve an even distribution of force during the testing procedures. Ten of these teeth formed the positive control group (Group 1), receiving no preparation. The other 50 teeth were prepared using a flat‐end fissure diamond bur (836/014; SS White Inc., Lakewood, NJ, USA) with a 1.4 mm diameter and a 6‐mm head length, mounted on a high‐speed handpiece with water and air spray for cooling. To prepare the teeth, the pulp chamber roof was removed, and a mesio‐occluso‐distal (MOD) cavity was created. The isthmus width of the occlusal cavity was set at two‐thirds of the intercuspal distance, while the gingival floor of the mesial and distal cavities was positioned 1 mm above the CEJ. The total depth of the occlusal preparation measured 6 mm from the pulpal floor to the cusp tips, with a proximal box buccolingual width of 4 mm. A low‐speed handpiece with a no. 6 carbide round bur (Brasseler USA, Savannah, GA, USA) was used to finalize the pulp chamber form (Figure 3a). The chamber was thoroughly cleaned with a spoon excavator to remove residual pulp tissue, rinsed with normal saline, and dried with air spray.

Among the prepared teeth, 10 specimens were designated as the negative control group (Group 2) and left unrestored. In the remaining 40 teeth, a 2‐mm layer of ZnO eugenol (ZOE) (Caulk‐Dentsply, Milford, DE, USA) was applied to the pulp chamber using moist cotton pellets. After completing the pulpotomy procedure, a metal matrix band was secured around each tooth with a Tofflemire retainer. The remaining 4 mm of each cavity was restored with RMGIC (Figure 3b), with the teeth divided into four experimental groups based on the additives incorporated:

Group 3: RMGIC

Group 4: RMGIC + 5 wt.% mesoporous ZnO NPs

Group 5: RMGIC + 1 wt.% BCNCs

Group 6: RMGIC + 5 wt.% mesoporous ZnO NPs and 1 wt.% BCNCs.

The preparation of RMGIC and the restorative procedures for these groups followed the same method described for the µSBS test. After completing the photopolymerization of the RMGIC, polishing was performed using Sof‐Lex Contouring and Polishing Discs (3 M ESPE, St. Paul, MN, USA), which are aluminum oxide‐coated flexible discs designed for contouring, finishing, and polishing composite restorations. This was followed by rubber polishers (Enhance Finishing and PoGo Polishing System, Dentsply Sirona, York, PA, USA), which are silicone‐based polishing instruments embedded with aluminum oxide particles, designed for final smoothing and high‐gloss polishing. All specimens were stored in distilled water at 37°C for 24 h.

For fracture resistance testing, each specimen was mounted on the lower part of a universal testing machine (Instron, Z020; Zwick Roell, Germany). A steel tip with a 4‐mm diameter was attached to the upper movable head of the device. A continuously increasing compressive force was applied parallel to the tooth's long axis at a speed of 0.5 mm/min (Figure 3c). The load was applied at the central fossa for all groups until fracture occurred (Figure 3d), except for Group 2, where it was directed at the midpoint of the buccolingual width. The force required to induce fracture was recorded in Newtons (N).

**Figure 4:**
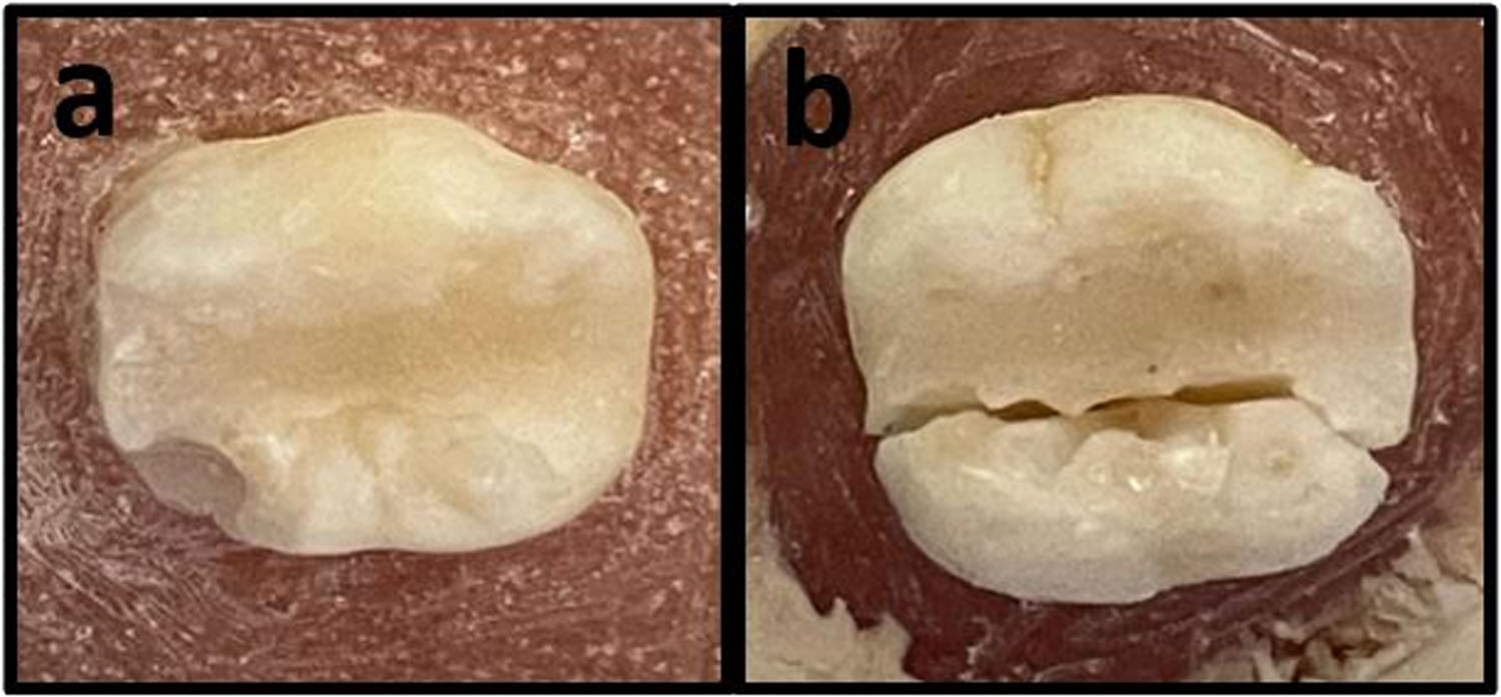
Classification of fracture patterns as restorable or unrestorable. (a) Restorable fractures. (b) Unrestorable fracture.

Fracture patterns were classified as either restorable or unrestorable. A fracture line above the simulated bone level (acrylic resin) was considered restorable (Figure 4a), while fractures extending below this level were deemed unrestorable (Figure 4b) (Topçuoğlu and Topçuoğlu 2023).

### 2.4 Statistical Analysis

Statistical analysis was conducted using SPSS software, version 20.0 (IBM SPSS, Chicago, IL, USA). A p‐value of less than 0.05 was considered statistically significant. The Kolmogorov‐Smirnov test was used to evaluate the normality of the data. To compare differences across groups, one‐way ANOVA was applied, followed by Tamhane's post hoc test for pairwise comparisons. Moreover, to assess the statistical significance of differences in fracture patterns, a Chi‐square test was conducted.

## 3 Results

**Figure 5:**
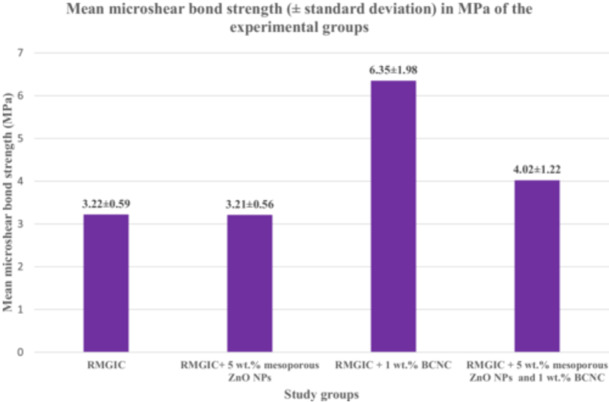
Bar graph illustrating the microshear bond strength values with their corresponding standard deviations across different study groups. (Abbreviations: BCNCs – bacterial cellulose nanocrystals; RMGIC – resin‐modified glass ionomer cement; ZnO NPs – zinc oxide nanoparticles.).

**Figure 6:**
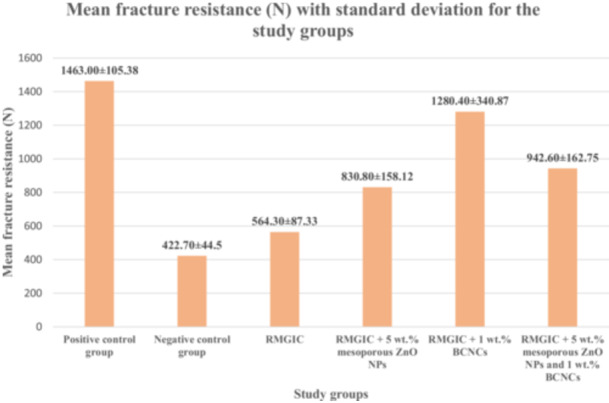
Bar graph displaying the mean fracture resistance values (in Newtons) along with the corresponding standard deviations for each study group. (Abbreviations: BCNCs – bacterial cellulose nanocrystals; RMGIC – resin‐modified glass ionomer cement; ZnO NPs – zinc oxide nanoparticles.).

The Kolmogorov‐Smirnov test confirmed that the mean µSBS and fracture resistance values of all study groups followed a normal distribution (*p* > 0.05). Table 1 summarizes the mean µSBS values and their standard deviations (SDs) in MPa, while Figure 5 presents these data in a bar chart. Similarly, Table 2 and Figure 6 illustrate the mean fracture resistance values (in Newtons) along with their respective SDs for all groups. Statistical analysis using one‐way ANOVA showed significant differences among the experimental groups for both µSBS and fracture resistance values (*p* < 0.001). To explore these differences further, a post hoc Tamhane test was performed.

**Table 1:** Mean microshear bond strength (MPa) with standard deviation for the experimental groups.

Group number	Group description	Mean micro shear bond strength ± standard deviation
1	RMGIC	3.22 ± 0.59^A^
2	RMGIC + 5 wt.% mesoporous ZnO NPs	3.21 ± 0.56^A^
3	RMGIC + 1 wt.% BCNCs	6.35 ± 1.98 ^B^
4	RMGIC + 5 wt.% mesoporous ZnO NPs and 1 wt.% BCNCs	4.02 ± 1.22^A^

*Note:* Different uppercase letters denote statistically significant differences between the groups as determined by the Tamhane test.

**Table 2:** Mean fracture resistance (N) with standard deviation for the study groups.

Group number	Group description	Mean fracture resistance ± standard deviation
1	Positive control group	1463.00 ± 105.38 ^A^
2	Negative control group	422.70 ± 44.50 ^B^
3	RMGIC	564.30 ± 87.33 ^C^
4	RMGIC + 5 wt.% mesoporous ZnO NPs	830.80 ± 158.12 ^D^
5	RMGIC + 1 wt.% BCNCs	1280.40 ± 340.87 ^AE^
6	RMGIC + 5 wt.% mesoporous ZnO NPs and 1 wt.% BCNCs	942.60 ± 162.75 ^DE^

*Note:* Different uppercase letters denote statistically significant differences between the groups as determined by the Tamhane test.

For µSBS, the Tamhane test revealed that the RMGIC + 1 wt.% BCNCs group achieved the highest mean µSBS value (6.35 ± 1.98), which was significantly higher than that of the control group (RMGIC) and the other experimental groups (*p* < 0.001). However, no statistically significant differences were detected among the remaining experimental groups (*p* > 0.05).

**Figure 7:**
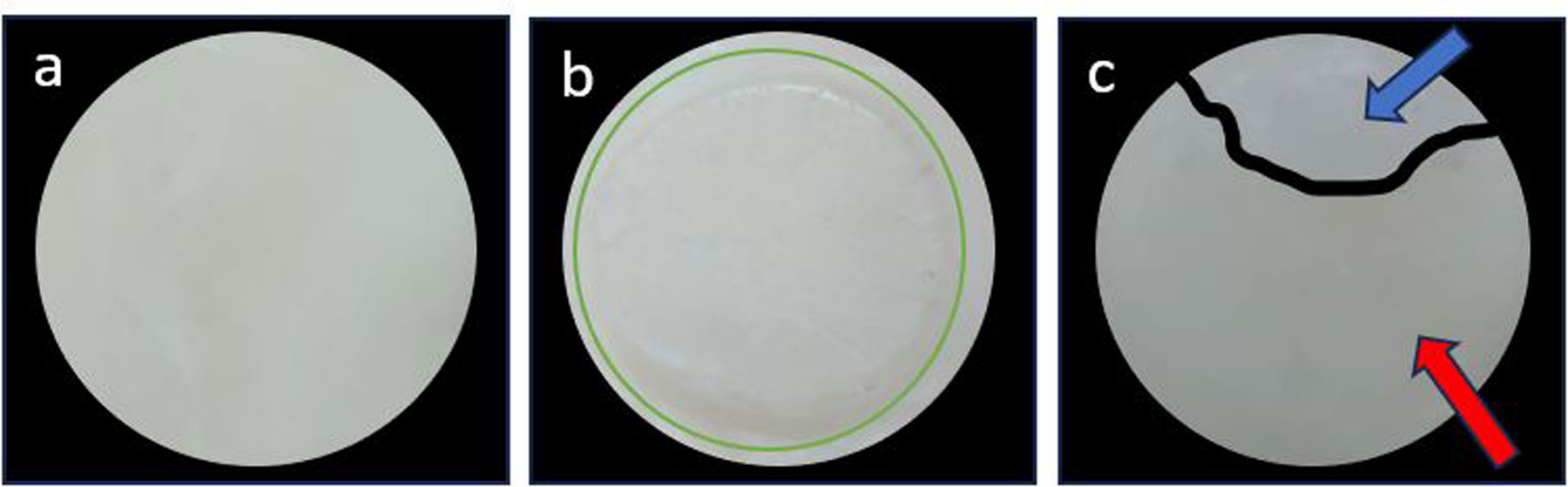
Various fracture modes observed under a stereomicroscope at 40× magnification during the microshear bond strength test. (a) Adhesive failure; (b) Cohesive failure within the resin‐modified glass ionomer cement (RMGIC), with the fracture area approximately outlined by a green circle; (c) Mixed failure, where cohesive failure in RMGIC is indicated by blue arrows, and adhesive failure is marked by red arrows.

**Figure 8:**
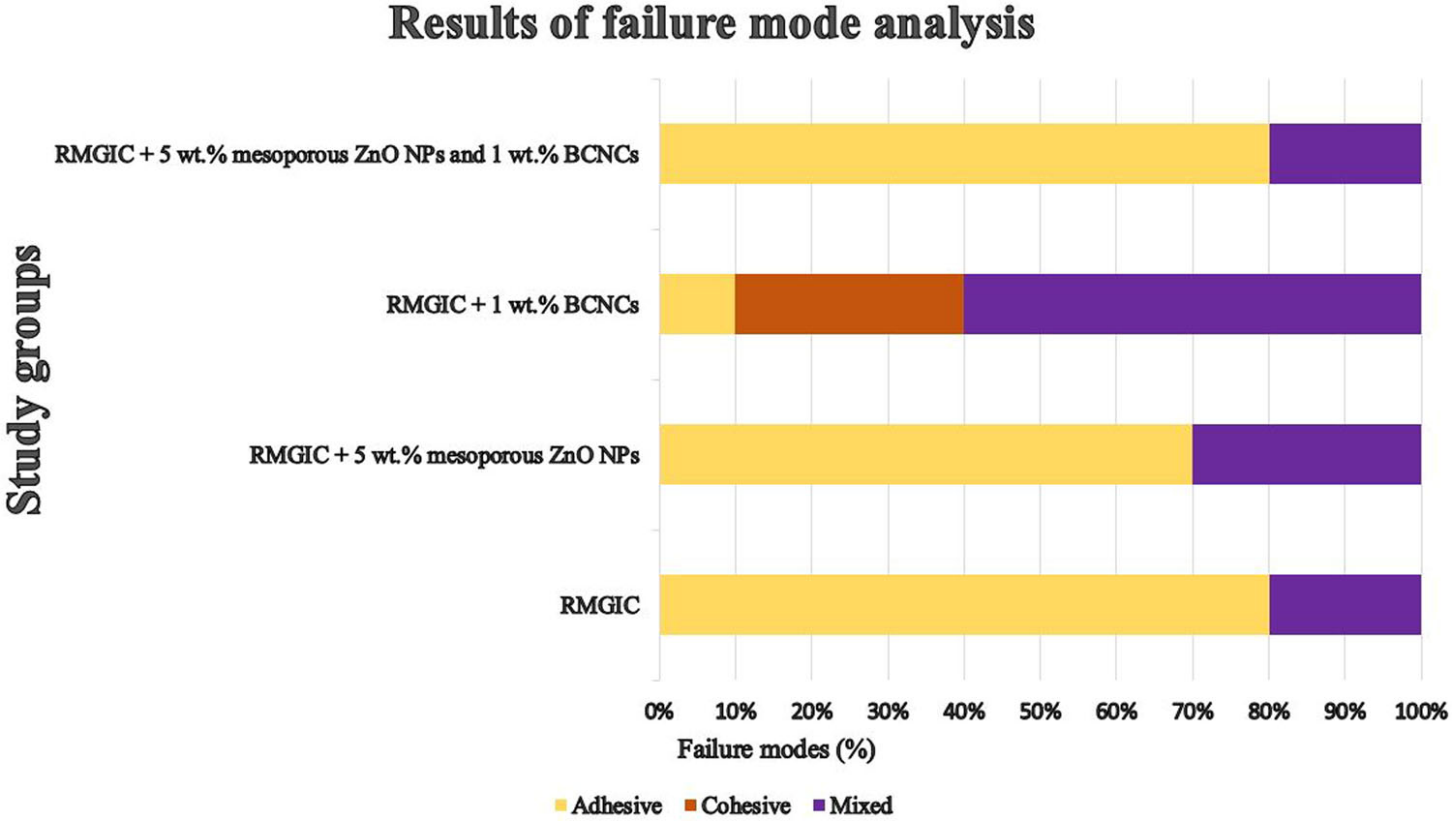
Bar chart depicting the failure mode analysis results for the microshear bond strength test. (Abbreviations: BCNCs – bacterial cellulose nanocrystals; RMGIC – resin‐modified glass ionomer cement; ZnO NPs – zinc oxide nanoparticles.).

Figure 7 shows representative failure modes observed during the µSBS test. Figure 8 displays the failure mode analysis results for the microshear bond strength test in the form of a chart. Adhesive failure was the most common mode in all groups, except for RMGIC + 1 wt.% BCNCs, which predominantly exhibited mixed failure. Cohesive failure within the RMGIC was observed in only three specimens of the RMGIC + 1 wt.% BCNCs group.

In terms of fracture resistance, the Tamhane test indicated that the negative control group had the lowest mean value (422.70 ± 44.50) among all groups, which was significantly lower than the other experimental groups (*p* < 0.05). The positive control group displayed the highest fracture resistance, significantly outperforming all other groups (*p* < 0.05), except for the RMGIC + 1 wt.% BCNCs group, which demonstrated comparable values (*p* > 0.05).

Fracture resistance in the RMGIC group was significantly lower than in all experimental groups, except for the negative control, which had a significantly lower value than RMGIC (all *p* < 0.05). The RMGIC + 5 wt.% mesoporous ZnO group exhibited significantly lower fracture resistance than the RMGIC + 1 wt.% BCNCs group but showed higher resistance compared to the RMGIC group (*p* < 0.05). No statistically significant difference was observed between the RMGIC + 5 wt.% mesoporous ZnO group and the RMGIC + 5 wt.% mesoporous ZnO NPs and 1 wt.% BCNCs group (*p* > 0.05).

The RMGIC + 1 wt.% BCNCs group exhibited significantly higher fracture resistance compared to both the RMGIC and RMGIC + 5 wt.% mesoporous ZnO groups (*p* < 0.05). Although the fracture resistance of the RMGIC + 1 wt.% BCNCs group (1280.40 ± 340.87) was greater than that of the RMGIC + 5 wt.% mesoporous ZnO NPs and 1 wt.% BCNCs group (942.60 ± 162.75), this difference was not statistically significant (*p* > 0.05).

**Figure 9:**
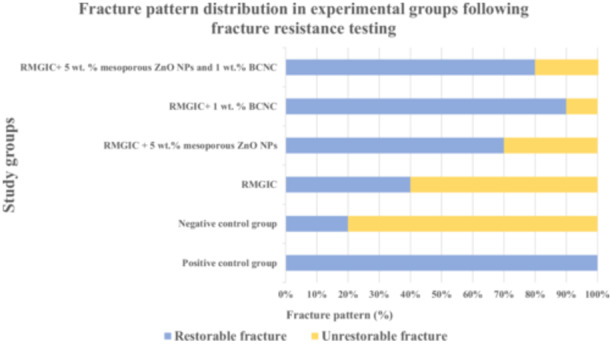
Bar chart showing the percentage distribution of fracture patterns classified as restorable or unrestorable across the study groups following the fracture resistance test. (Abbreviations: BCNCs – bacterial cellulose nanocrystals; RMGIC – resin‐modified glass ionomer cement; ZnO NPs – zinc oxide nanoparticles.).

Figure 9 displays the percentage distribution of fracture patterns classified as restorable or unrestorable across the study groups following the fracture resistance test. Fracture pattern analysis revealed variation in the distribution of restorable and unrestorable fractures across the study groups. As shown in Figure 9, the positive control group exhibited 100% restorable fractures, indicating high fracture resistance. In contrast, the negative control group had only 20% restorable fractures, suggesting lower fracture resistance. The RMGIC group demonstrated 40% restorable fractures, while the RMGIC + 5 wt.% mesoporous ZnO NPs group showed 70%, and the RMGIC + 1 wt.% BCNCs group displayed 90% restorable fractures. The combination of RMGIC with both 5 wt.% mesoporous ZnO NPs and 1 wt.% BCNCs resulted in 80% restorable fractures. To assess the statistical significance of these differences, a Chi‐square test was performed (*p* < 0.001), indicating a significant difference in fracture pattern distribution among the study groups. These results indicate that higher fracture resistance, especially in the RMGIC + 1 wt.% BCNCs group, corresponds with a greater proportion of restorable fractures, whereas the negative control group exhibited more unrestorable fractures.

A Pearson correlation analysis was conducted to evaluate the relationship between fracture resistance and µSBS. The analysis revealed a moderate positive correlation between these two variables (Pearson correlation coefficient = 0.667), which was statistically significant (*p* < 0.001). This finding suggests that higher microshear bond strength values are associated with greater fracture resistance within the study groups.

## 4 Discussion

This study assessed the µSBS of RMGIC to primary dentin and the fracture resistance of primary molars restored with RMGIC, with or without mesoporous ZnO NPs and BCNCs. Incorporating 1 wt.% BCNCs significantly enhanced both µSBS and fracture resistance compared to RMGIC alone, with fracture resistance comparable to intact teeth, highlighting its reinforcing potential. The addition of 5 wt.% mesoporous ZnO moderately improved fracture resistance.

The µSBS test, a reliable method for measuring bond strength, allows multiple specimens from a single tooth and better evaluates small surfaces (Sadat‐Shojai et al. 2010). Unlike macroshear tests, which may produce uneven stress and mixed loading modes, µSBS minimizes these limitations. In macroshear tests, low applied loads often cause cohesive failure within dentin rather than at the adhesive interface (Sadat‐Shojai et al. 2010). Therefore, µSBS was used to examine the effects of mesoporous ZnO NPs and BCNCs on RMGIC bond strength to primary dentin.

Fracture resistance testing is essential for evaluating the mechanical durability of restored teeth, as it simulates occlusal forces encountered in the oral environment and assesses the material's ability to withstand functional loads without catastrophic failure (Shafiei et al. 2020; Selvaraj et al. 2023). Compared to conventional compressive strength tests, it provides a more clinically relevant evaluation by considering cavity preparation, material adaptation, and adhesive interactions (Kaur et al. 2022). It also differentiates between restorable and non‐restorable fractures, critical for predicting long‐term clinical success (Topçuoğlu and Topçuoğlu 2023). Primary molars, subjected to dynamic and repetitive occlusal loads, require restorations with high fracture resistance to ensure longevity and reduce premature failure (Topçuoğlu and Topçuoğlu 2023). Therefore, assessing the fracture resistance of modified RMGIC restorations provides important evidence for their potential clinical application in pediatric dentistry.

Antibacterial properties of restorative materials are crucial, as bacteria like Streptococcus mutans can persist after decay removal, and microscopic gaps may increase secondary caries risk (Moradpoor et al. 2021). GICs exhibit antibacterial effects mainly from fluoride release, though their low pH during setting may contribute more (Hafshejani et al. 2017). However, post‐setting antibacterial activity is uncertain, and secondary caries remains one of the primary causes of GIC failure, indicating fluoride alone may be insufficient (Hafshejani et al. 2017). This limitation highlights the need to enhance GICs' antimicrobial properties, often by incorporating additional antibacterial agents (Jowkar et al. 2019; Hafshejani et al. 2017).

ZnO NPs have attracted significant attention in dentistry for their strong antibacterial properties, which are enhanced by their high surface area, biocompatibility, stability, affordability, and low toxicity (Moradpoor et al. 2021; Demir et al. 2014). ZnO also binds strongly to polyacrylic acid in RMGIC, and adding 2 wt.% ZnO NPs provides antimicrobial benefits without compromising flexural strength or fluoride release (Malekhoseini et al. 2021).

Mesoporous NPs, including calcium‐silicate and silica, offer additional therapeutic potential, providing controlled chlorhexidine release, strong antibacterial effects against E. faecalis and L. casei, low cytotoxicity, remineralization properties, and suitability for bone defect repair and intra‐canal medications, without compromising material integrity (Fan et al. 2016; Zhang et al. 2014). Mesoporous ZnO NPs, recognized for their antibacterial properties, biodegradability, and drug‐release capabilities, have potential as antibacterial additives in RMGIC (Jowkar et al. 2025, 2024; Laurenti et al. 2020). Their mesoporous structure enhances surface area, improving antibacterial efficiency (Jowkar et al. 2025). While reducing bacterial adhesion, it is crucial that NPs do not impair bond strength or fracture resistance, which this study evaluated in primary teeth restored with RMGIC containing mesoporous ZnO NPs. Moreover, to improve the mechanical performance of RMGIC, this study evaluated the use of BCNCs as a reinforcing filler (Moradian et al. 2021a).

The present study showed that adding 1 wt.% BCNCs to RMGIC significantly improved both µSBS and fracture resistance compared to the control, with fracture resistance comparable to intact teeth. Based on previous studies, 1 wt.% BCNCs was chosen to optimize mechanical properties. Silva et al. reported that 1 wt.% CNCs enhanced compressive strength, abrasion resistance, and bond strength of GICs without affecting disintegration, setting time, or solubility, while higher concentrations caused NP aggregation and reduced performance (Menezes‐Silva et al. 2017). Similarly, Moradian et al. found that RMGIC with 1 wt.% BCNCs exhibited higher SBS to permanent dentin, and other studies confirmed improved µSBS to primary dentin at this concentration (Moradian et al. 2021b; Mohammadi et al. 2023). The enhanced mechanical performance of the RMGIC + 1 wt.% BCNCs group in the present study is likely due to strong interactions between BCNCs and the cement matrix. BCNCs form hydrogen bonds with glass hydroxyl groups and polyacrylic acid carboxyl groups, improving adhesion and material integrity (Moradian et al. 2021a). Their nanoscale size ensures uniform dispersion, reduces defects, fills gaps, and provides additional bonding sites for polymer chains. Swelling in polyacrylic acid further strengthens chemical bonds with calcium and phosphate ions in dentin (Menezes‐Silva et al. 2019; Silva et al. 2013; Moradian et al. 2021a, 2021b). These mechanisms create an interconnected BCNCs network that reinforces the cement, impedes crack propagation, and enhances load distribution through hydrogen bonding and electrostatic interactions (Moradian et al. 2021a). Together, these synergistic effects confirm BCNCs as an effective reinforcing additive, supporting both bond strength and durability in pediatric restorations.

This study also evaluated the effects of incorporating 5 wt.% mesoporous ZnO NPs into RMGIC, based on previous findings that this concentration optimally enhances GIC reactivity (Titien Hary Agustantina et al. 2024). The addition moderately improved fracture resistance and bond strength to primary dentin, though less than the BCNCs group, yet still exceeded the control (RMGIC alone). The small size, large surface area, and ordered mesoporous structure of ZnO NPs likely fill gaps, enhance resin matrix interaction, and reinforce micromechanical bonding (Bai et al. 2020; Samuel et al. 2009). Potential chemical interactions with RMGIC may further improve particle integration and uniformity, contributing to enhanced fracture resistance and material adaptation at the adhesive interface (Malekhoseini et al. 2021). The mesoporous structure of ZnO NPs likely improved material adaptation at the adhesive interface, enhancing bond strength. Although their reinforcement effect was less than BCNCs, ZnO NPs still increased fracture resistance compared to the control, providing a balanced improvement in antibacterial and mechanical properties without compromising bond strength or structural integrity, making them a promising RMGIC additive.

Another key finding of this study was that the RMGIC + 5 wt.% mesoporous ZnO NPs and 1 wt.% BCNCs group did not show a significant improvement in bond strength compared to the RMGIC control, indicating that the combined additives did not notably enhance adhesion to dentin. However, in fracture resistance, this group outperformed the control and fell between the RMGIC + 1 wt.% BCNCs and RMGIC + 5 wt.% mesoporous ZnO groups, although the difference with the ZnO group was not statistically significant. These results suggest that while combining ZnO NPs and BCNCs can improve mechanical properties, it does not exceed the reinforcement achieved by BCNCs alone. This may reflect a saturation effect, where excessive NPs agglomerate, disperse unevenly, and create weak points. Clustering can interfere with integration into the RMGIC matrix, while higher NP content may reduce Al³⁺ availability for crosslinking with polyacrylic acid and limit polymer interactions, compromising fracture resistance and bond strength (Horszczaruk et al. 2014; De Caluwé et al. 2017). These findings emphasize the need to optimize NP concentration to balance antimicrobial benefits with mechanical performance.

Failure mode analysis showed adhesive failures predominated in all groups except RMGIC + 1 wt.% BCNCs, which mainly exhibited mixed and cohesive failures, indicating a stronger internal structure. Cohesive fractures often correlate with higher bond strength, supporting the superior adhesion of BCNCs‐reinforced RMGIC (Fatima et al. 2024). The interconnected BCNCs network likely enhanced mechanical properties, improving fracture resistance and interfacial bonding. In contrast, the other groups exhibited predominantly adhesive failures, whereas the combination of 5 wt.% mesoporous ZnO NPs with 1 wt.% BCNCs modified the fracture behavior, underscoring the unique reinforcing effect of BCNCs on the structural integrity of RMGIC.

Fracture pattern analysis reflected the effect of material modifications on fracture resistance. The positive control exhibited only restorable fractures, while the negative control mostly showed unrestorable ones. In experimental groups, RMGIC alone had 40% restorable fractures, increasing to 70% with 5 wt.% mesoporous ZnO NPs and 90% with 1 wt.% BCNCs. The combination of both reached 80%, indicating improved resistance but not exceeding BCNCs alone. These results confirm that BCNCs strengthens the cement matrix, reducing adhesive failures and enhancing fracture resistance, while slightly lower performance of the combined group may result from NP agglomeration, emphasizing the need for optimized formulations.

Pearson correlation analysis revealed a significant moderate positive correlation between fracture resistance and µSBS, indicating that higher bond strength generally contributes to improved fracture resistance. This finding aligns with failure mode and fracture pattern analyses, where groups with higher bond strength, especially RMGIC + 1 wt.% BCNCs, showed greater fracture resistance and a higher proportion of restorable fractures. The moderate correlation suggests that other factors, such as material composition and NP dispersion, also affect fracture resistance, highlighting the need for further studies to optimize RMGIC formulations. These results are particularly relevant to pediatric dentistry, where primary tooth restorations must endure dynamic occlusal forces.

This study has some limitations. Only one commercially available RMGIC was tested, and bond strength was assessed after 24 h of water storage, without evaluating long‐term durability. Being in vitro, the study cannot fully replicate the oral environment, including pH fluctuations, chewing forces, and brushing effects. Moreover, the dispersion of mesoporous ZnO NPs and BCNCs within the RMGIC matrix was not verified using microscopic or spectroscopic techniques. Although manual and mechanical mixing were performed, NP agglomeration remains possible, which could influence mechanical outcomes. Future studies should incorporate microscopic or spectroscopic analyses to confirm homogeneity and optimize mixing protocols. Future studies should confirm NP homogeneity, test different concentrations, evaluate long‐term performance, and examine additional mechanical and physical properties to better understand the impact of these additives on RMGIC.

## 5 Conclusion

Incorporating 1 wt.% BCNCs into RMGIC effectively enhances both dentin bond strength and fracture resistance in primary molars. The addition of mesoporous ZnO NPs provided moderate improvements in mechanical performance but was less effective than BCNCs. These findings suggest that BCNCs‐modified RMGIC has potential for producing more durable and reliable restorations in pediatric dentistry, offering improved structural integrity and higher chances of restorable fractures. Further studies are needed to optimize NP concentrations and evaluate long‐term clinical performance.

## Author Contributions

**Ali Nozari:** conceptualization, data curation, methodology, supervision, writing – review. **Farnaz Haji Abbas Oghli:** conceptualization, data curation, methodology, supervision, writing – review. **Fatemeh Parvizi:** conceptualization, data curation, methodology, supervision, writing – review. **Zahra Jowkar:** conceptualization, data curation, formal analysis, funding acquisition, investigation, methodology, project administration, resources, software, supervision, validation, visualization, roles/writing – original draft, and writing – review and editing. **Maryam Pakniyat Jahromi:** conceptualization, data curation, methodology, supervision, writing – review. **Seyed Ahmadreza Hamidi:** conceptualization, data curation, methodology, supervision, writing – review.

## Conflicts of Interest

The authors declare no conflicts of interest.

## Data Availability

Data available on request from the author.
